# Disaggregation of Hepatobiliary Cancer Mortality Among Asian Americans: Analysis of NVSS Mortality Data

**DOI:** 10.1002/cam4.71259

**Published:** 2025-09-29

**Authors:** Anna Park, Andrew Vodinh‐Ho, Ivory Rok, Xinran Qi, George A. Hung, Nicholas Kikuta, Armaan Jamal, Gloria S. Kim, Latha P. Palaniappan, Malathi Srinivasan, Robert J. Huang, Adrian M. Bacong

**Affiliations:** ^1^ School of Medicine Georgetown University Washington DC USA; ^2^ Department of Physics Princeton University Princeton New Jersey USA; ^3^ Department of Medicine University of Nevada, Reno School of Medicine Reno Nevada USA; ^4^ Department of Epidemiology Johns Hopkins Bloomberg School of Public Health Baltimore Maryland USA; ^5^ Department of Medicine Rutgers New Jersey Medical School Newark New Jersey USA; ^6^ Center for Asian Health Research and Education Stanford University School of Medicine Stanford California USA; ^7^ Department of Medicine Johns Hopkins University School of Medicine Baltimore Maryland USA; ^8^ Division of Cardiovascular Medicine Stanford University School of Medicine Stanford California USA; ^9^ Division of Primary Care and Population Health Stanford University School of Medicine Stanford California USA; ^10^ Division of Gastroenterology and Hepatology Stanford University School of Medicine Stanford California USA

**Keywords:** Asian Americans, extrahepatic cholangiocarcinoma, gallbladder cancer, hepatobiliary cancer, hepatocellular carcinoma, intrahepatic cholangiocarcinoma, mortality

## Abstract

**Background:**

Asian Americans (AAs) are a diverse population, and aggregation of AA health data in national reports conceals significant differences between AA subgroups. As hepatobiliary cancer rates increase globally, a greater understanding of hepatobiliary mortality among AA subgroups could motivate precision intervention and screening programs.

**Methods:**

Using national mortality data from 2005 to 2020, we report age‐adjusted mortality rates, standardized mortality ratios, and annual percent change for hepatocellular carcinoma (HCC), nonspecified liver cancer (NOS), intrahepatic cholangiocarcinoma (ICC), extrahepatic cholangiocarcinoma (ECC), and gallbladder cancer (GBC) using national mortality data for the six largest AA subgroups (Asian Indian, Chinese, Filipino, Japanese, Korean, and Vietnamese) compared to non‐Hispanic White people (NHW).

**Results:**

All AA subgroups (except Asian Indians) had significantly higher hepatobiliary cancer mortality than NHW people. Vietnamese people demonstrated the highest mortality from HCC (7.65 per 100,000) and nonspecified liver cancer (5.57 per 100,000), while Korean people had the highest mortality from the biliary tract cancers: ICC (3.10 per 100,000), GBC (0.72 per 100,000), and ECC (0.97 per 100,000). Notably, ICC mortality increased across the study period. Across all subgroups, male individuals had significantly higher hepatobiliary cancer mortality than female individuals, with differences being largest for HCC and nonspecified liver cancer.

**Conclusions:**

Differences in mortality across hepatobiliary cancer types demonstrate the importance of analyzing subtypes separately. These differences also highlight the importance of developing ethnically targeted screening, prevention strategies, and treatment.

## Graphical abstract

Aggregation of cancer health data for Asian Americans conceals potential high‐risk groups within this diverse population. Using mortality records from 2005 to 2020, we find considerable heterogeneity in liver and biliary cancer subtypes by disaggregated Asian subgroup when compared to non‐Hispanic White decedents.

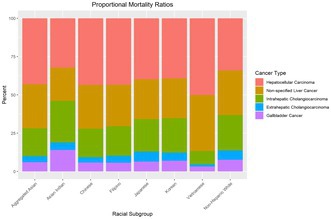

**Abbreviations**
AA: Asian American
ACS: American Community Survey
AMR: age‐adjusted mortality rate per 100,000 individuals
APC: annual percent change
CDC: Centers for Disease Control and Prevention
CI: confidence interval
ECC: extrahepatic cholangiocarcinoma
GBC: gallbladder carcinoma
HBsAg: hepatitis B surface antigen
HBV: hepatitis B virus
HCC: hepatocellular carcinoma
HCV: hepatitis C virus
ICC: intrahepatic cholangiocarcinoma
ICD‐10: International Classification of Diseases, 10th revision
NAFLD: nonalcoholic fatty liver disease
NHW: non‐Hispanic White
NOS: nonspecified liver cancer
SMR: standardized mortality ratio
US: United States
USPSTF: US Preventive Services Task Force

## 1 Introduction

Asian Americans (AAs) are the fastest‐growing racial/ethnic group in the United States (US), comprising about 7% of the nation's overall population, and projected to surpass 46 million people by 2060 [1]. This growing population comprises more than 20 countries of origin in East Asia, Southeast Asia, and the Indian subcontinent [1]. The diversity of the AA population is reflected in an immense diversity in culture, language, genetics, socioeconomic status, and healthcare access in America. Despite the significant heterogeneous characteristics of Asian subgroups, epidemiological studies in the US have historically aggregated the Asian population [2, 3], concealing trends and data in health outcomes and preventing a detailed understanding of subgroup‐specific risks that would allow for more precise intervention and screenings.

AAs are the only racial or ethnic group in the US in which cancer is the leading cause of death in both men and women [4]. By contrast, cardiovascular diseases are the leading cause of death in all other racial groups [4]. Aggregation of health data among the AA population may perpetuate the idea that AA people have either lower or comparable disease risk profiles to non‐Hispanic White people [5]. However, disaggregation of AAs has revealed heterogeneous health profiles for multiple diseases, including obesity [6], type 2 diabetes [6, 7, 8], and cardiovascular disease [8, 9, 10, 11, 12].

Hepatobiliary cancers, encompassing liver and biliary tract cancers, are increasing both in the US and globally [4, 13, 14, 15]. Liver and biliary tract cancers are named after where the cancer originates in the body. Liver cancer is a heterogeneous cancer starting in the liver, with the most common type being hepatocellular carcinoma (HCC). Biliary tract cancers affect the tracts that drain (biliary tree) and temporarily store (gallbladder) liver and pancreatic metabolic waste and digestive enzymes. These cancers include intrahepatic cholangiocarcinoma (ICC), extrahepatic cholangiocarcinoma (ECC), and gallbladder carcinoma (GBC) [16]. Liver cancer is the fastest increasing cause of cancer mortality in the US, with an annual rise of 1.7% per year from 2009 to 2018 [15]. Both ICC and ECC mortality rose in all racial groups during the same time period [17]. These cancers are often difficult to diagnose early because of asymptomatic and heterogeneous appearance in early cancer stages [18], lower sensitivity of detection with ultrasound, lack of routine collection of cancer biomarkers (e.g., alpha‐fetoprotein) [19], and underutilization of surveillance in the US when compared to countries with higher rates of liver cancer [20]. Additionally, liver cancers are characterized by a poor prognosis for patients affected [4]. AAs are especially affected by liver diseases and liver cancer, partly due to high rates of chronic hepatitis B virus (HBV) infection that is endemic to many Asian countries of origin. Although only comprising 7% of the population, AAs disproportionately experience around 60% of all HBV burden in the US [5]. HBV infection has also been identified as a risk factor for ICC, along with hepatitis C virus (HCV) infection [21, 22]. Other risk factors include chronic inflammatory diseases of the liver, such as liver cirrhosis, nonalcoholic fatty liver disease (NAFLD), and primary sclerosing cholangitis [22, 23, 24, 25]. Recent studies have found associations of cholangiocarcinoma with these risk factors [23]. While the different risk factors for liver cancers and biliary cancers highlight the need to distinguish between them, most existing literature categorizes both as liver cancer.

Although there have been epidemiologic studies examining disaggregated mortality data for liver cancer among AAs [24, 25, 26, 27, 28], this study provides insight into the importance of studying hepatobiliary cancers separately. This study uses the NVSS mortality dataset from 2005 to 2020 to define trends between racial subgroups and hepatobiliary mortality rates to determine the burden of each cancer type on death in AAs.

## 2 Methods

### 2.1 Study Data and Population

The National Vital Statistics System (NVSS) Mortality Database provides detailed mortality records in the US from 2005 to 2020, including the year of death, age of death, sex, race, ethnicity, and underlying cause of death for each decedent [29]. Given that this study is a secondary data analysis of publicly available, de‐identified mortality data, this study was exempted by the Stanford University School of Medicine Institutional Review Board.

The International Classification of Disease, 10th revision (ICD‐10) codes were used to define the underlying cause of death based on death certificates. These codes defined the hepatobiliary cancers included in this study. Liver cancers included HCC (C22.0) and nonspecified liver cancer (NOS liver—C22.9), while biliary tract cancers included ICC (C22.1), ECC (C24.0, C24.8, and C24.9), and GBC (C23). “All hepatobiliary cancer” mortality rates were generated by combining all of these groups.

We included all decedents that were identified in mortality records as non‐Hispanic Asian Indian, Chinese, Filipino, Japanese, Korean, and Vietnamese, and used the non‐Hispanic White (NHW) population as the reference group for analysis. Race in mortality records was based on reports from close relatives to decedents, linkage of mortality records to birth records, or by physician determination. These six AA subgroups were chosen for analysis because they collectively make up more than 85% of the Asian population in America [3, 30]. Any AA decedent that identified as multiple races or was categorized as “Other Asian” was excluded from the analysis. Additionally, Pacific Islanders were excluded from the analysis.

### 2.2 Calculation of Mortality Rates

To calculate mortality rates (i.e., number of decedents for a cause of death divided by the underlying population strata), we used the NVSS Mortality Dataset to generate numerator data for each cancer subtype by race, sex, and 10‐year age category. We used annual estimates derived from the American Community Survey (ACS) to create population denominators, also stratified by race, sex, and 10‐year age category. The ACS is an annual survey conducted by the US Census that obtains social, economic, housing, and demographic information across the nation that influences the development of programs and policies [31]. ACS data from 2005 to 2020 were used as denominator data to approximate AA subgroup populations for each individual year by race, sex, and age.

### 2.3 Statistical Analysis

Direct age standardization was weighted according to the 2010 US Census estimated population. Specifically, for each given year (e.g., 2020), we calculated sex‐and age‐stratum‐specific mortality rates for a given cancer subtype and race group. We then applied the 2010 US Census estimated population as a weight to adjust for differences in age distribution between the given race group and the US population. We then combined the age‐specific rates to calculate an overall age‐standardized mortality rate (AMR) for the given year. Age standardization was done to account for the confounding of age demographics with race. For example, the Japanese‐American population is generally older than the NHW and other AA populations.

We analyzed three outcomes stratified by sex: AMRs, proportional standardized mortality ratios (SMRs) with 95% confidence intervals (CIs), and trends over time using annual percent changes (APCs) and average annual percent changes (AAPCs). AMRs were calculated as deaths per 100,000 people following the method mentioned above. SMRs were calculated as the ratio of the AMRs of a specific AA subgroup divided by the rate of the reference NHW population. Data cleaning and analysis of AMRs and SMRs were performed using R version 4.3.1.22 [32].

Trends over time and APCs were used to identify the direction and magnitude of mortality trends from 2005 to 2020 using Joinpoint Regression Version 5.0.2 [33]. Joinpoint Regression also allows us to evaluate possible nonlinear trends in cancer mortality by determining key inflection points where APCs change from 2005 to 2020. These inflection points (i.e., joinpoints) represent key years where mortality trends shift direction (e.g., increase vs. decrease). Inflection points are determined by first starting with a minimum number of inflection points (e.g., 0 inflection points or a straight line) and increasing the number of inflection points to determine the model of best fit. Best‐fitting models are determined using the Akaike information criterion and Bayesian information criterion. Statistical significance of trends is determined using the Monte Carlo Permutation method [33]. In our results, we provide both AAPC, which represents the general change in mortality from 2005 to 2020, as well as highlight individual APCs during unique time periods where yearly mortality rates shift.

## 3 Results

### 3.1 All Hepatobiliary Cancers and Overall Cancer Burden

Over the 16‐year study period, we observed 758,289 AA and 33,231,057 NHW deaths. From these, we identified 19,337 AA deaths and 282,935 NHW deaths from hepatobiliary cancer, accounting for 2.6% and 0.9% of all deaths among AAs and NHWs, respectively. Hepatobiliary cancer deaths constituted 1.4% of Asian Indian, 3.0% of Chinese, 2.0% of Filipino, 1.6% of Japanese, 3.9% of Korean, and 5.2% of Vietnamese deaths. The total number of hepatobiliary decedents and total population per racial subgroup from 2005 to 2020 is shown in Table 1, stratified by both age group and sex. For all racial subgroups, over 70% of hepatobiliary cancer decedents were over 60 years old. For all subgroups except Japanese, over half of hepatobiliary cancer decedents were male.

**TABLE 1:** Number of hepatobiliary cancer decedents and total population for non‐Hispanic White, Aggregated Asian American, and Disaggregated Asian American group by age and sex, 2005–2020 National Vital Statistics System.

	Aggregated Asian		Asian Indian		Chinese		Filipino	
	Total decedents (%)	Total population (%)	Total decedents (%)	Total population (%)	Total decedents (%)	Total population (%)	Total decedents (%)	Total population (%)
Age group								
0–39	360 (1.8)	111 137 857 (52.4)	34 (2.3)	32,466,906 (62.8)	133 (2.1)	29 409 739 (51.0)	60 (1.7)	19 513 232 (47.3)
40–59	4141 (20.8)	62 240 682 (29.4)	328 (22.0)	13,143,146 (25.4)	1359 (21.5)	17 159 632 (29.8)	670 (19.5)	12 972 268 (31.4)
60+	15,436 (77.4)	38 560 154 (18.2)	1131 (75.8)	6,091,967 (11.7)	4831 (76.4)	11 091 505 (19.2)	2706 (78.8)	8 800 941 (21.3)
Sex								
Male	12,622 (63.3)	100 274 083 (47.3)	930 (62.3)	26,925,422 (52.1)	4243 (67.1)	27 036 775 (46.9)	2020 (58.8)	18 163 411 (44.0)
Female	7315 (36.7)	111 664 610 (52.7)	563 (37.7)	24,746,597 (47.9)	2080 (32.9)	30 624 101 (53.1)	1416 (41.2)	23 123 030 (56.0)
	Japanese		Korean		Vietnamese		Non‐Hispanic White	
	Total decedents (%)	Total population (%)	Total decedents (%)	Total population (%)	Total decedents (%)	Total population (%)	Total decedents (%)	Total population (%)
Age Group								
0–39	144 (7.4)*	4 019 395 (33.0)	40 (1.4)	11,674,378 (51.6)	84 (2.2)	14 054 207 (53.0)	2051 (0.7)	1 443 888 155 (45.8)
40–59		3 938 374 (32.4)	625 (21.5)	6,926,530 (30.6)	1024 (26.7)	8 100 732 (30.6)	55,003 (19.4)	893 827 269 (28.4)
60+	1789 (92.6)	4 207 067 (34.6)	2247 (77.2)	4,036,532 (17.8)	2732 (71.2)	4 362 142 (16.5)	225,881 (79.8)	815 454 893 (25.9)
Sex								
Male	829 (42.9)	5 150 270 (42.3)	1703 (58.5)	10,159,733 (44.9)	2897 (75.4)	12 838 472 (48.2)	177,877 (62.9)	1 551 964 725 (49.2)
Female	1104 (57.1)	7 014 566 (57.7)	1209 (41.5)	12,477,707 (55.1)	943 (24.6)	13 679 609 (51.6)	105,058 (37.1)	1 601 205 592 (50.8)

* The 0–39 age group was merged with the 40–59 group as it had fewer than 10 decedents. Note that “Total Population” represents the sum of the total population of the given race group from 2005 to 2020.

**FIGURE 1:**
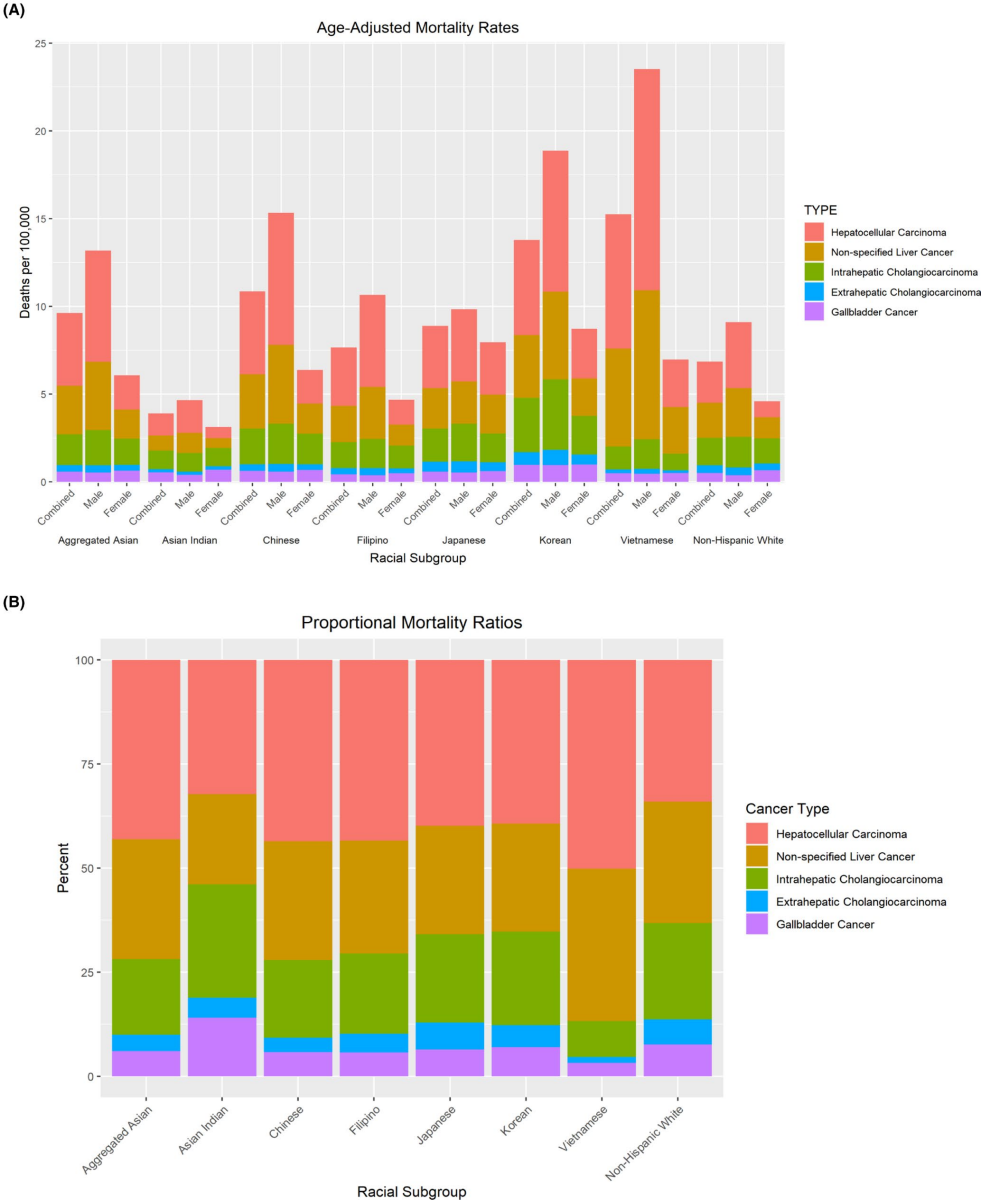
(A) Age‐standardized mortality rate (per 100,000 people) from hepatobiliary cancers for non‐Hispanic White, Asian Americans in aggregate, and disaggregated Asian American group, by sex, (B) Proportional mortality ratio from hepatobiliary cancers by race, 2005–2020 National Vital Statistics System.

Figure 1A shows the age‐adjusted mortality rate of each racial subgroup for all hepatobiliary cancers in aggregate and each separate cancer disaggregated by sex. Table S1 reports the age‐adjusted mortality rates and SMR in tabular format. Figure 1B shows the proportional mortality by race group. Nearly all AA subgroups demonstrated significantly higher hepatobiliary cancer mortality than NHW people (6.85 deaths per 100,000 people). Notably, Vietnamese (15.24 per 100,000) and Korean (13.79 per 100,000) populations demonstrated the highest mortality from hepatobiliary cancers, more than twofold higher than NHW people. Interestingly, Asian Indian people (3.91 deaths per 100,000) had significantly lower hepatobiliary cancer mortality than all of the other AA subgroups and NHW people. This trend was consistent for both Asian Indian females and males.

With respect to proportional mortality, HCC comprised the majority of hepatobiliary cancer deaths among each race group, with proportional mortality being highest among the Vietnamese. Additionally, nonspecified liver cancer was higher among Vietnamese individuals when compared to other racial and ethnic groups.

### 3.2 Hepatocellular Carcinoma

In aggregate, AAs (4.14 per 100,000) demonstrated higher mortality from HCC than NHW people (2.33 per 100,000). Additionally, all AA subgroups besides Asian Indians (SMR = 0.54, 95% CI 0.50–0.58) had increased mortality compared to NHWs. Vietnamese (SMR = 3.29, 95% CI 3.14–3.43), Korean (SMR = 2.33, 95% CI 2.20–2.46), and Chinese (SMR = 2.03, 95% CI 1.95–2.11) people had between two and three‐fold higher HCC mortality when compared to NHW people. Figure 1B depicts the proportional mortality ratios of each hepatobiliary cancer type for each racial subgroup. The proportional mortality ratio of HCC was highest for the Vietnamese group. There were significant differences in mortality between sexes, with males having between 1.38 and 4.65 times higher mortality than females across all racial subgroups.

When analyzing males separately, differences in mortality across racial subgroups are similar to those for the general population. Vietnamese (SMR = 3.34, 95% CI 3.18–3.51), Korean (SMR = 2.13, 95% CI 1.99–2.28), and Chinese males (SMR = 2.00, 95% CI 1.91–2.09) had up to threefold higher mortality than NHW males (3.77 per 100,000). However, analyzing female mortality rates had a different trend. Japanese (SMR = 3.34, 95% CI 2.91–3.81), Korean (SMR = 3.17, 95% CI 2.85–3.51), Vietnamese (SMR = 3.04, 95% CI 2.74–3.36), and Chinese (SMR = 2.17, 95% CI 2.00–2.35) females demonstrated higher mortality than NHW females (0.89 per 100,000).

**FIGURE 2:**
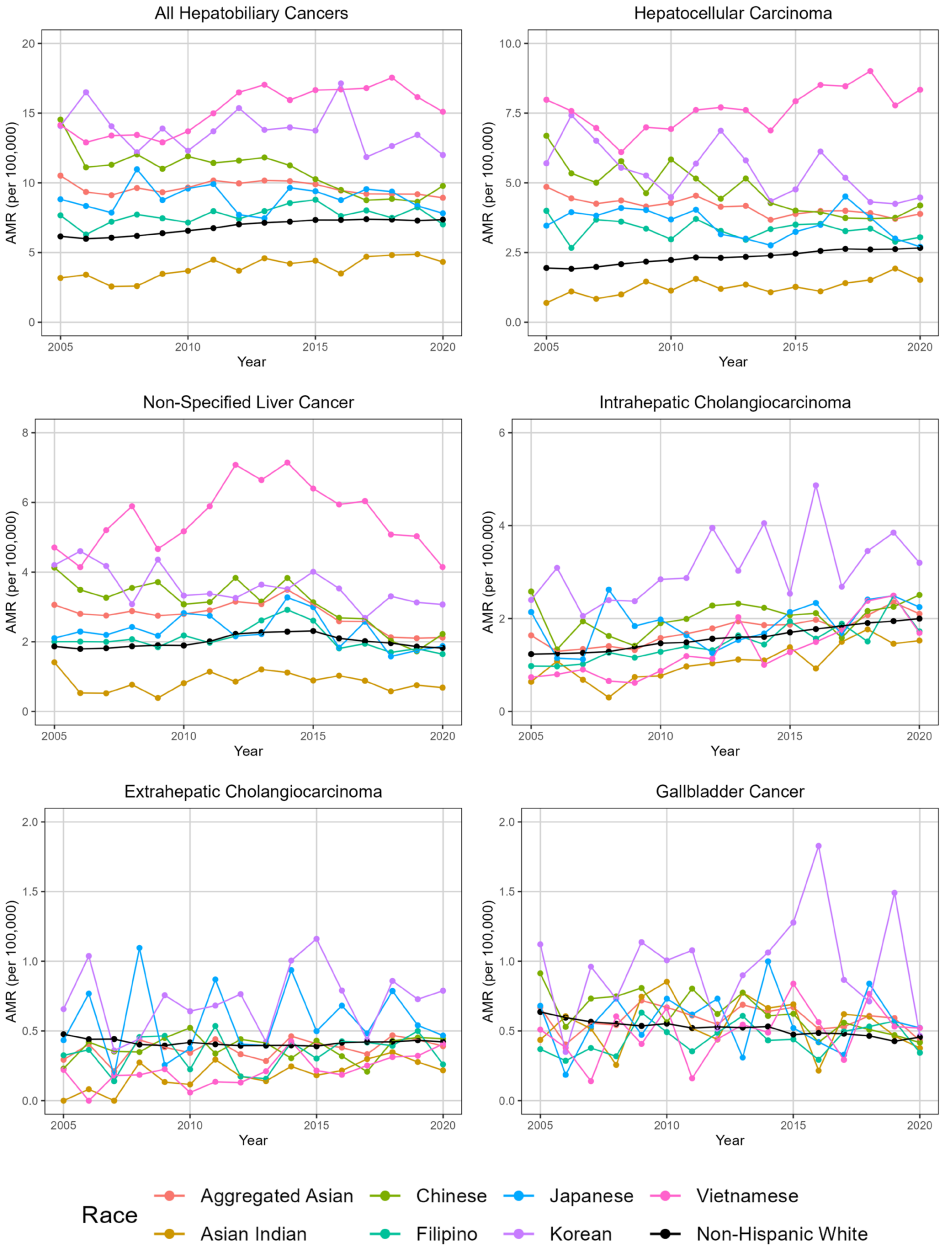
Annual age‐adjusted mortality rates (per 100,000 people) from all hepatobiliary cancers (Panel A), hepatocellular carcinoma (Panel B), nonspecified liver cancer (Panel C), intrahepatic cholangiocarcinoma (Panel D), extrahepatic cholangiocarcinoma (Panel E), and gallbladder cancers (Panel F). The data are presented for non‐Hispanic White people (NHWs) and disaggregated Asian American subgroups for the years 2005–2020.

Figure 2 depicts mortality trends as AAPC from 2005 to 2020 for both sexes in aggregate derived using Joinpoint Regression (tabular results in Table S2). AA people demonstrated a significant decrease in HCC mortality (AAPC = −1.0%, 95% CI −1.3, −0.5%), while NHW people demonstrated a significant increase (AAPC = 2.4%, 95% CI 2.0, 2.9%). However, differences emerge when analyzing AA subgroups separately (Figure 2B). Some AA groups, such as Koreans, had decreased HCC mortality (AAPC = −2.3%, 95% CI −3.3, −0.6%); other groups, such as Asian Indian (AAPC = 3.4%, 95% CI 2.0, 5.5%) and Vietnamese (AAPC = 1.4%, 95% CI 0.7, 2.5%) people demonstrated significant increases in mortality over the study period. However, other groups experienced fluctuations in mortality rates despite general declines in HCC mortality from 2005 to 2020 (Table S3 for tabular results). Japanese (AAPC = −2.8%, 95% CI −3.7, −1.7%) and Chinese (AAPC = −2.5%, 95% CI −3.5, −1.4%) are two groups that had an overall decline in HCC but with fluctuations in mortality. Japanese people, for example, experienced a general decline in HCC mortality from 2005 to 2014 (Average Percent Change (APC) = −3.3%, 95% CI −12.0, −1.0%). However, from 2014 to 2017, Japanese people had a dramatic uptick in HCC mortality (APC = 11.8%, 95% CI 0.9, 18.7%), only to experience another decline from 2017 to 2020 (APC = −14.0%, 95% CI −21.3, −7.4%). In comparison, Chinese people had a general decline from 2005 to 2018 (APC = −3.8%, 95% CI −9.9, −2.4%), only to experience a slight increase, albeit not statistically significant, from 2018 to 2020 (APC = 6.4%, 95% CI = −1.4, 13.4%). Mortality rates stratified by sex are shown in Figure S2 (Males) and Figure S3 (Females) with tabular results in Table S3.

When stratified by sex, only Asian Indian males (AAPC = 4.2%, 95% CI 2.4, 6.9%) and Vietnamese males (AAPC = 1.5%, 95% CI 0.6, 2.8%) experienced increases in HCC mortality, while most other AA groups experienced declines in HCC mortality (Table S2). Among females, Filipinos (AAPC = 2.1%, 95% CI = 0.6, 5.1%) and Vietnamese experienced an increase in HCC mortality (AAPC = 1.5%, 95% CI 0.4, 4.8%). Interestingly, Chinese females had a general decline in HCC mortality from 2005 to 2018 (APC = −5.6%, 95% CI −10.7, −3.3%), followed by an increase from 2018 to 2020 (APC = 19.2%, 95% CI 3.2, 32.5%). However, overall, Chinese females had a 2.6% decline in HCC mortality from 2005 to 2020 (95% CI −4.0, −0.9%). Additionally, Korean women appeared to have a nonsignificant decline in mortality (AAPC = −4.9%, 95% CI −6.7, 2.7). However, while there was a nonsignificant decline from 2005 to 2018 (APC = −2.5%, 95% CI −11.4, 76.8%), there was a sharp decline from 2018 to 2020 (APC = −19.1%, 95% CI −27.9, −1.0%).

### 3.3 Nonspecified Liver Cancer

As with HCC, AA people (2.77 per 100,000) demonstrated significantly higher mortality from NOS liver cancer than NHW people (2.00 per 100,000). Of the AA subgroups, only Asian Indian (SMR = 0.42, 95% CI 0.38–0.46) and Filipino (SMR = 1.04, 95% CI 0.97–1.11) people did not have higher mortality than NHW people. Vietnamese people (SMR = 2.79, 95% CI 2.65–2.93) demonstrated the highest mortality relative to NHW people. The proportional mortality ratio of NOS liver cancer was also highest for the Vietnamese group, potentially indicating that most liver cancer deaths that were not specified were actually HCC. Across all racial subgroups except for Japanese, male individuals had higher mortality than female individuals.

Differences in mortality for male individuals are similar to those for both sexes in aggregate. Asian Indian people (SMR = 0.41, 95% CI 0.37–0.46) demonstrated lower mortality than NHW people, while mortality for both Filipino (SMR = 1.06, 95% CI 0.98–1.16) and Japanese (SMR = 0.87, 95% CI 0.72–1.03) people did not differ significantly from NHW mortality. Vietnamese males (SMR = 3.05, 95% CI 2.87–3.24) had the highest mortality relative to NHW males. Results are similar for females, with all groups besides Asian Indian (SMR = 0.45, 95% CI 0.38–0.53) and Filipino (SMR = 0.98, 95% CI 0.87–1.10) females having higher mortality than NHW females, and Vietnamese (SMR = 2.18, 95% CI 1.96–2.41) females having the highest mortality.

From 2005 to 2020 (Figure 2C), AA people (AAPC = −1.8%, 95% CI −2.3, −0.5%) demonstrated a general decrease in mortality (Table S2). However, one Joinpoint was noted in 2014 (Table S3). From 2005 to 2014, AA people experienced a nonsignificant increase in NOS liver cancer (APC = 1.5%, 95% CI −0.2%, 5.4%). However, from 2014 to 2020, AA people experienced a significant decline in NOS liver cancer (APC = −6.5%, 95% CI −8.6%, −4.1%). In comparison, mortality did not change significantly for NHW people (AAPC < 0.1%, CI −0.4%, 0.3%). However, this was due in part to a generally flat trend from 2005 to 2010 (APC = 0.8%, 95% CI −2.3%, 2.1%), with an increase from 2010 to 2019 (APC = 7.6%, 95% CI 4.2%, 9.3%), followed by a decline from 2013 to 2020 (APC = −3.7%, 95% CI −4.5%, −3.0%). When disaggregated by Asian subgroup (Figure 2C), Asian Indian, Chinese, and Korean individuals experienced a general decline in NOS liver cancer. In comparison, Filipinos, Japanese, and Vietnamese had a generally flat AAPC. However, this lack of change in AAPC may be partially explained by a major inflection point in NOS liver cancer mortality rates around 2014 (Figure 2). Prior to 2014, Filipinos (APC = 3.5%, 95% CI 1.1%, 9.4%) and Vietnamese (APC = 5.9%, 95% CI 2.9%, 12.3%) experienced an increase in NOS liver cancer mortality. However, from 2014 to 2020, Filipinos (APC = −6.9%, 95% CI −10.0%, −37.0%) and Vietnamese (APC = −8.7%, 95% CI −11.7%, −4.9%) had a decline in NOS liver cancer mortality. Similar trends were seen among Japanese people (2005–2014 APC = 2.3%, 95% CI −5.0%, 51.2%; 2014–2020 APC = −6.1%, 95% CI −15.7%, 2.6%), but these trends were not statistically significant.

When disaggregated by sex, AA males experienced a general decline in NOS liver cancer mortality (AAPC = −1.5%, 95% CI −1.9%, −0.8%) while AA females did not experience a significant change in NOS liver cancer mortality (AAPC = −2.1%, 95% CI −3.1%, 1.5%). However, similar to our general results, AA females experienced a nonsignificant increase from 2005 to 2014 (APC = 1.3%, 95% CI −1.4%, 27.9%) followed by a decline from 2014 to 2020 (APC = −7.0%, 95% CI −12.8%, −3.2%).

### 3.4 Intrahepatic Cholangiocarcinoma

AA people (1.75 per 100,000) demonstrated significantly higher ICC mortality than NHW people (1.58 per 100,000). However, the Asian Indian (SMR = 0.67, CI 0.62–0.73) and Vietnamese (SMR = 0.83, 95% CI 0.75–0.92) people had lower mortality than NHW people, while Filipino people's mortality (SMR = 0.93, 95% CI 0.86–1.01) was not significantly different compared to NHW people. Alternatively, Japanese (SMR = 1.20, 95% CI 1.05–1.35) and Chinese (SMR = 1.28, 95% CI 1.21–1.35) people had higher mortality, and Korean people had the highest mortality (SMR = 1.96, 95% CI 1.82–2.11). Similar to the other liver cancers, males had significantly higher mortality than females across all racial subgroups, excluding Asian Indian people.

Results are similar for males compared to the population as a whole, with AA males (2.01 per 100,000) demonstrating higher mortality than NHW males (1.74 per 100,000). Of the AA subgroups, Japanese (SMR = 1.24, 95% CI 1.02–1.48), Chinese (SMR = 1.33, 95% CI 1.22–1.43), and Korean (SMR = 2.31, 95% CI 2.09–2.54) males had higher mortality than NHW males, with the difference between Koreans and NHWs being the largest. Among females, similar but smaller differences emerged. While Asian Indian (SMR = 0.75, 95% CI 0.66–0.84) and Vietnamese (SMR = 0.67, 95% CI 0.56–0.79) females had lower mortality than NHW females, Chinese (SMR = 1.22, 95% CI 1.12–1.32) and Korean (SMR = 1.56, 95% CI 1.36–1.73) females had significantly higher mortality than NHW females.

For both AA people (APC 3.0%, 95% CI 2.1, 4.5%) and NHW people (APC 3.5%, 95% CI 3.3, 3.7%), ICC mortality significantly increased over the study period (Figure 2D). All AA subgroups experienced increases in mortality over the study period, although the increase in Korean people's mortality (AAPC 1.7%, 95% CI −0.2, 4.5%) was not statistically significant (Table S2). In particular, Vietnamese (AAPC 6.2%, 95% CI 4.0, 9.3%) and Asian Indian (AAPC 6.9%, 95% CI 4.3, 10.9%) people's mortality had the sharpest increases over the study period. None of the regressions had any Joinpoints (Table S3), demonstrating that the increase in mortality was consistent across the study period for all AA subgroups and NHWs.

Trends are fairly similar between males and females. For males, all AA subgroups and NHWs had increases in ICC mortality over the study period, with the increase not being statistically significant for Koreans only. Again, no Joinpoints were included in any of the regressions. Similarly, for females, all AA subgroups and NHWs generally had increases in mortality over the study period, although these increases were not statistically significant for the Chinese and Japanese groups.

### 3.5 Extrahepatic Cholangiocarcinoma

Of all of the hepatobiliary cancers, ECC is the only one for which AA mortality (0.38 per 100,000) was lower than NHW mortality (0.42 per 100,000). Although AA mortality was low overall, there were clear disparities between subgroups. While most subgroups had either similar or low ECC mortality compared to NHW people, Japanese (SMR = 1.38, 95% CI 1.07–1.72) and Korean (SMR = 1.73, 95% CI 1.47–2.00) people had significantly higher mortality. As with the liver cancers, male mortality (0.42 per 100,000) was higher than female mortality (0.33 per 100,000) for AA people.

Besides this difference in overall mortality, there were no significant differences in mortality between the males and females when looking at racial subgroups individually. For both sexes, Korean (male SMR = 1.95, 95% CI 1.56–2.38, female SMR = 1.48, 95% CI 1.15–1.84) people had significantly higher mortality than NHW (male = 0.44 per 100,000, female = 0.39 per 100,000) people.

Due to the relatively low number of ECC deaths, trends over time in mortality were mostly insignificant (Figure 2E). Over the course of the study period, AAs as a whole and AA subgroups did not have any statistically significant AAPCs. On the other hand, NHWs had a statistically significant decrease in mortality over the study period (AAPC = −0.7%, 95% CI −1.1, 0.0%, Table S2). This decrease was mostly driven by a sharp decrease in mortality between 2005 and 2009 (APC = −4.3%, 95% CI −10.1, −1.2%), which was followed by a slight increase from 2009 to 2020 (APC = 0.7%, 95% CI 0.1, 2.1%) (Table S3). The only other subgroup with a Joinpoint in the regression was the Vietnamese group, who experienced a nonsignificant decrease in mortality from 2005 to 2010 (APC = −16.7%, 95% CI −52.6, 5.7%), followed by a significant increase from 2010 to 2020 (APC = 13.7%, 95% CI 9.9, 44.1%).

Only Vietnamese (AAPC = 8.4%, 95% CI 4.5, 16.4%) and Chinese (AAPC = 2.6%, 95% CI 0.5, 10.9%) males had significant AAPCs. While the trend was steady across the study period for Vietnamese males, Chinese males experienced an insignificant increase in mortality from 2005 to 2010 (APC = 9.0%, 95% CI −1.8, 116.4%) followed by a decrease from 2010 to 2016 (APC = −10.3%, 95% CI −32.6, −3.2%) and another increase from 2016 to 2020 (APC = 16.5%, 95% CI 6.7, 59.1%). NHW males had no significant change over the study period, but they saw a significant decrease in mortality between 2005 and 2013 (APC = −1.5%, 95% CI −5.1, −0.2%) followed by an increase between 2013 and 2020 (APC = 2.2%, 95% CI 0.9, 5.9%). AA females as a whole experienced a significant increase in mortality over the study period (AAPC = 1.9%, 95% CI 0.1, 4.8%), which was driven by significant increases experienced by Asian Indian (AAPC = 6.0%, 95% CI 1.7, 14.2%), Korean (AAPC = 4.6%, 95% CI 2.2, 9.7%), and Vietnamese (AAPC = 2.9%, 95% CI 2.2, 9.7%) females. On the other hand, mortality decreased for NHW females (AAPC = −1.3%, 95% CI −1.8, −0.4%). This was driven by a sharp decrease from 2005 to 2009 (APC = −5.1%, 95% CI −11.4, 1.4%).

### 3.6 Gallbladder Carcinoma

While low, aggregated AA people (0.59 per 100,000) had higher GBC mortality than NHW people (0.52 per 100,000). Of the AA subgroups, only Filipino (SMR = 0.84, 95% CI 0.73–0.97) people had significantly lower mortality than NHW people, while Chinese (SMR = 1.21, 95% CI 1.09–1.34) and Korean (SMR = 1.86, 95% CI 1.62–2.11) people had higher mortality. While males generally have higher hepatobiliary cancer mortality, AA females (0.65 per 100,000) had higher gallbladder cancer mortality than their male counterparts (0.52 per 100,000).

Results for males individually are similar but more pronounced. While most subgroups had similar mortality to NHW people, Chinese (SMR = 1.55, 95% CI 1.32–1.80) and Korean (SMR = 2.52, 95% CI 2.04–3.05) people had significantly higher mortality, with the difference between Korean and NHW people being especially magnified. As a whole, female AAs (0.65 per 100,000) had similar gallbladder cancer mortality compared to that of NHW people (0.66 per 100,000). Filipino (SMR = 0.75, 95% CI 0.62–0.89) and Vietnamese (SMR = 0.78, 95% CI 0.61–0.97) females had low mortality compared to NHW females, while only Korean (SMR = 1.48) females had significantly higher mortality.

Over the study period, mortality only decreased significantly for the Chinese (AAPC = −4.5%, 95% CI −5.7, −2.8%; Table S2) and NHW (AAPC = −2.0%, CI −2.5, −1.5%) subgroups (Figure 2F). However, AAs as a whole experienced a sharp decrease in mortality from 2018 to 2020 (APC = −16.0%, 95% CI −22.2, −2.3%). Furthermore, Koreans experienced an increase in mortality from 2005 to 2016 (APC = 6.8%, 95% CI 0.3, 56.0%) followed by a decrease from 2016 to 2020 (APC = −22.2, 95% CI −42.1, −7.2%).

For males, only Asian Indians (AAPC = 4.8%, 95% CI 0.5, 16.5%) and NHWs (AAPC = −1.6%, CI −2.2, −0.9%) experienced significant changes in mortality across the study period. The Asian Indian trend was driven by a sharp increase in mortality from 2005 to 2010 (APC = 40.3%, 95% CI 10.8, 208.3%, Table S3), which was followed by a decrease from 2010 to 2020 (APC = −9.4%, 95% CI −15.4, −3.4%). For females, NHWs again had a significant decrease in mortality across the study period (AAPC = −2.7%, 95% CI −3.1, −2.0%), while neither AAs in aggregate nor any AA subgroups had significant changes in mortality.

## 4 Discussion

In our analysis of US death records from 2005 to 2020, we examined hepatobiliary cancer mortality trends among disaggregated AA subgroups in comparison to NHWs. Previous research has reported distinct and divergent temporal patterns of mortality rates between AA subgroups for GI cancers, including esophageal, gastric, colorectal, liver, and pancreatic cancers [33]. Notably, AA subgroups—particularly Vietnamese, Chinese, Japanese, Filipino, and Korean Americans—experience higher liver cancer mortality compared to NHW people during earlier timeframes, from 2003 to 2017 [33]. Our study expands upon these findings by further segregating hepatobiliary cancers by anatomical subtypes—HCC, ICC, ECC, gallbladder cancer (GBC), and nonspecified liver cancer (NOS). This subtype‐level examination uncovered substantial heterogeneity in mortality rates across AA subgroups. We find evidence that hepatobiliary cancer generally increased among both NHW and AA groups from 2005 to 2020. Additionally, in congruence with previous studies [4], we find evidence that mortality from ICC has increased over time while ECC has remained stable with heterogeneity in trends by disaggregated AA group.

Given that these subtypes have unique etiologies and environmental risk factors, more precise identification of hepatobiliary cancer subtypes can help guide targeted public health interventions and screening modalities, therapeutic strategies, and improve patient outcomes. Mortality from hepatobiliary cancer varies nearly threefold between high mortality (e.g., Vietnamese and Korean American people) and low mortality (e.g., Asian Indian people) subgroups. Moreover, the distribution of hepatobiliary cancer mortality demonstrates substantial variability among AA subgroups, underscoring the imperative need to develop national strategies for mitigating cancer risk and prevention, as well as treatment tailored to the diverse, multiethnic populations of the US.

Cancer mortality incorporates both cancer incidence and measures of survival after diagnosis. The high and increasing incidence, coupled with the poor prognosis of liver cancer and disparities in risk factors and care, results in a strong association between the number of new cases and the number of deaths [34, 35]. High hepatobiliary cancer mortality among certain populations may indicate higher cancer incidence, differences in cancer screening, and/or lower cancer survival. Notably, Vietnamese people had the highest mortality from HCC (7.65 per 100,000) and nonspecified liver cancer (5.57 per 100,000). In contrast, Korean Americans suffered the highest mortality from biliary tract cancers: ICC (3.10 per 100,000), GBC (0.72 per 100,000), and ECC (0.97 per 100,000), exceeding not only other AA subgroups but also NHW counterparts.

HCC development is influenced by a range of factors, including chronic infections with HBV and HCV infections, alcoholic liver disease, hemochromatosis, and nonalcoholic steatohepatitis, alongside genetic and environmental contributions [34, 36]. Among AA populations, HBV infection has been identified as a predominant risk factor for HCC, particularly in Chinese, Korean, Hmong, Vietnamese, Cambodian, and Laotian individuals, whereas HBV is much less prevalent among Japanese and South Asian populations, whose liver cancer profiles more closely resemble those of NHWs [37]. The predominance of HBV‐related HCC across AA subgroups underscores the need for culturally tailored screening and prevention strategies as well as treatment availability and efficacy. Our finding of elevated HCC mortality in Vietnam is consistent with prior work that has documented a significant upward trend in liver cancer mortality among Vietnamese men [33].

Consistent with prior literature, we have found that in AA subgroups, male individuals experienced significantly higher hepatobiliary cancer mortality rates compared to females [33]. This difference was most pronounced for HCC and nonspecified liver cancer. As previously reported, liver cancer accounted for over 20% of all cancer deaths among Vietnamese men and approximately 10% among Vietnamese women. These findings emphasize the intersection of sex‐ and ethnicity‐specific risk, underscoring the need for sex‐responsive prevention, screening strategies, and treatment in high‐risk populations. Moreover, national trends reported in JAMA show that between 1999 and 2020, mortality from liver and intrahepatic bile duct cancers increased among both men and women across all US census regions [38]. Our results align with these broader trends while highlighting how the burden is unevenly distributed across AA subgroups, particularly among Vietnamese men.

Our analysis of trends over time reveals the heterogeneity in diverging temporal patterns of mortality among AA subgroups. Vietnamese people, for example, experienced a general increase in HCC from 2005 to 2020. However, during that same time period, Vietnamese people experienced a dramatic increase in nonspecified liver cancer from 2005 to 2014, only to experience a general decline from 2015 to 2020. Despite these heterogeneous trends, Vietnamese people continued to have some of the highest cancer mortality rates among disaggregated AA subgroups. The high burden of increasing mortality rates from liver cancer among Vietnamese Americans may likely be associated with the high prevalence of chronic HBV infection (estimated at > 10%) in Vietnam, when comparing infection rates to the general American population (estimated at < 1%) [39]. Additionally, liver cancer mortality has been noted to be sharply increasing in Vietnam and has recently become one of the leading causes of death, surpassing lung cancer [40]. As most Vietnamese people immigrated to the US after 1975 [41], the communal trajectory of increasing liver cancer between US‐born Vietnamese Americans and immigrant‐born Vietnamese likely reflects the vertical transmission patterns of chronic hepatitis B from mother to child [42].

Joinpoint regression analysis revealed important temporal trends in hepatobiliary cancer mortality. While HCC remains the most prevalent subtype, ICC mortality rates increased across every racial and ethnic group during the study period. This consistent upward trend in ICC underscores a shifting landscape in liver cancer burden and reinforces previous findings documenting rising ICC incidence and mortality in the US [4]. Despite being historically less common than HCC, ICC is gaining prominence as a key driver of hepatobiliary cancer deaths. The mechanisms behind this rise remain unclear but may involve increasing prevalence of risk factors such as chronic liver inflammation (i.e., cirrhosis and chronic HBV/HCV infection) [43], biliary tract infections (i.e., primary sclerosing cholangitis, biliary cysts, and stones) [44], parasitic infections [45], metabolic conditions (i.e., NAFLD, obesity, type 2 diabetes mellitus) [46], other inflammatory conditions (i.e., inflammatory bowel disease and chronic pancreatitis), and toxins (alcohol consumption and tobacco smoking) alongside improved detection and diagnostic classification [42]. These findings highlight the need for more focused epidemiologic and clinical research on ICC, as well as targeted interventions to address this emerging trend within the broader context of liver cancer prevention, especially in high‐risk populations.

### 4.1 Limitations and Strengths

It is important to acknowledge the limitations in our analysis. First, race and ethnicity information collected from death certificates follows the classification standards set by the Office of Management and Budget (OMB) to ensure consistent reporting across the US [47, 48]. This information is typically provided by the funeral director, who may have obtained this information from an informant, such as a family member or individual who was familiar with the deceased. Additionally, for multiracial individuals, the NVSS employs a process called “bridging,” which reassigns multirace responses to single‐race categories to facilitate consistent reporting and ease of calculation of vital statistics [49]. While this method serves practical purposes, it may obscure the true racial or ethnic identities of multiracial individuals. The extent of such misclassification is unknown and warrants further investigation. Implications of misclassification can introduce bias, particularly for groups that are more likely to be assigned a different AA group than the one with which they would self‐identify. Moreover, individuals with partial Asian heritage may be classified into an Asian subgroup they do not personally identify with, or are excluded from Asian categories altogether. In our analysis, AA decedents that were classified as multiracial or “Other Asian” were not included in our analyses to allow for more accurate comparisons between US data with established cultural patterns and disease epidemiology in the country of origin.

Similarly, classification of causes of death poses its own difficulties. These data are collected from decedents who may have comorbidities and are recorded by physicians, medical examiners, or funeral directors based on known clinical knowledge or information provided by next of kin or observation [35, 48, 49]. Death certificates in the United States include sections for the immediate cause of death, the underlying cause of death, and other significant conditions contributing to death. Physicians are required to complete these sections, detailing the sequence of events leading to death and any comorbid conditions [49]. Errors in medical certification, such as reporting ill‐defined conditions or incorrect sequencing of causes, can impact the accuracy of the underlying cause of death. It may be possible that an individual who died of hepatobiliary cancer was misattributed to dying of other causes, which may have been potentially exacerbated by or related to their cancer diagnosis. This misattribution—whether due to overlapping symptoms, complications, or clinical judgment—can result in underreporting of hepatobiliary cancer deaths, potentially skewing our findings. Furthermore, hepatobiliary cancer mortality may be imprecise because the larger NOS category encompasses HCC, ICC, and malignant neoplasms of the liver.

Covariates such as HBV infection, alcohol consumption, stage of cancer at diagnosis, or access to healthcare were not considered. As previous literature suggests that hepatobiliary carcinomas may be associated with these environmental risk factors, which may differ between the US and Asian countries, future research on these related factors is warranted. Additionally, coding for biliary tract cancers may be imprecise, as the nonspecified (NOS) category is quite large and encompasses malignant neoplasms of the liver that are not specified as primary or secondary malignancies.

Finally, although Joinpoint Regression allows us to evaluate possible nonlinear trends in cancer mortality by identifying key inflection points where the APCs change from 2005 to 2020, there are some inherent limitations. First, Joinpoint Regression is unable to incorporate additional covariates that may confound trends in hepatobiliary cancer over time. Additionally, Joinpoint regression simply describes trends in cancer mortality but cannot identify factors (e.g., medical innovations, public health interventions, policy changes) that underlie changes in trends. Finally, the accuracy of these trends also relies on the data quality, the time span of years examined, and model assumptions. Future work should examine how trends in hepatobiliary cancer are related to changes in medical innovations, public health interventions, and policy changes.

Nonetheless, our study is novel in presenting unique data that pertains specifically to cultivating a baseline understanding of hepatobiliary cancer subtypes. However, aligned with prior research findings, we demonstrate strong sex‐ and race/ethnicity‐specific patterns in mortality rates of hepatobiliary cancer among AA subgroups. Notably, previous studies have shown that liver cancer constituted over 20% of Vietnamese male cancer deaths, higher than in any other racial group [15, 36]. Liver cancer also constituted approximately 10% of Vietnamese female cancer deaths, the leading cause of GI cancer death in that subgroup [15, 36]. While Vietnamese individuals bore a disproportionate burden of liver cancer mortality, Chinese, Japanese, Filipinos, and Koreans also exhibited elevated mortality rates among both sexes compared to NHWs [4, 15, 33].

## 5 Conclusion

Our study contributes to the broader understanding of health disparities within AA subgroups within the US. By highlighting hepatobiliary cancer mortality rates across different AA subgroups, we provide valuable insights into the complex interplay of sociodemographic, cultural, and biological factors influencing disease outcomes. This nuanced understanding can inform targeted public health interventions aimed at reducing disparities and improving health equity among these populations. Additionally, our study underscores the importance of disaggregated data analysis in epidemiological research. By disaggregating data by AA subgroups, we were able to uncover disparities that may have been obscured in aggregated data collection and analyses. This approach allows for more precise identification of at‐risk populations and facilitates the development of tailored interventions to address their specific needs and risk factors.

## Author Contributions

**Anna Park:** conceptualization (equal), data curation (equal), methodology (equal), visualization (equal), writing – original draft (equal). **Andrew Vodinh‐Ho:** conceptualization (equal), data curation (equal), formal analysis (equal), methodology (equal), visualization (equal), writing – original draft (equal), writing – review and editing (equal). **Ivory Rok:** conceptualization (equal), data curation (equal), formal analysis (equal), methodology (equal), visualization (equal), writing – original draft (equal), writing – review and editing (equal). **Xinran Qi:** conceptualization (equal), supervision (equal), writing – review and editing (equal). **George A. Hung:** supervision (equal), writing – review and editing (equal). **Nicholas Kikuta:** supervision (equal), writing – review and editing (equal). **Armaan Jamal:** supervision (equal), writing – review and editing (equal). **Gloria S. Kim:** formal analysis (equal), supervision (equal), writing – review and editing (equal). **Latha P. Palaniappan:** funding acquisition (equal), supervision (equal), writing – review and editing (equal). **Malathi Srinivasan:** resources (equal), supervision (equal), writing – review and editing (equal). **Robert J. Huang:** conceptualization (equal), methodology (equal), resources (equal), supervision (equal), writing – review and editing (equal). **Adrian M. Bacong:** formal analysis (equal), methodology (equal), supervision (equal), writing – review and editing (equal).

## Conflicts of Interest

The authors declare no conflicts of interest.

## Supporting information


**Figure S1:** Age‐standardized proportional mortality ratio from hepatobiliary cancers for NHWs, AAs in aggregate, and each AA subgroup.

**Figure S2:** Annual age‐adjusted mortality rates (per 100,000) for males from all hepatobiliary cancers, hepatocellular carcinoma, non‐specified liver cancer, intrahepatic cholangiocarcinoma, extrahepatic cholangiocarcinoma, and gallbladder cancers. The data is presented for non‐Hispanic Whites (NHWs) and disaggregated Asian American subgroups for years 2005 to 2020.

**Figure S3:** Annual age‐adjusted mortality rates (per 100,000) for females from all hepatobiliary cancers, hepatocellular carcinoma, non‐specified liver cancer, intrahepatic cholangiocarcinoma, extrahepatic cholangiocarcinoma, and gallbladder cancers. The data is presented for non‐Hispanic Whites (NHWs) and disaggregated Asian American subgroups for years 2005 to 2020.

**Table S1:** Age‐standardized mortality rate (per 100,000 people) and standardized mortality ratios (SMR) from hepatobiliary cancers by race: 2005–2020 National Vital Statistics System.

**Table S2:** Average annual percent change (AAPC) in age‐standardized mortality from hepatobiliary cancers for non‐Hispanic White, aggregated Asian American, and disaggregated Asian American groups, 2005–2020 National Vital Statistics System.

**Table S3:** Annual percent change (APC) joinpoint regression results of age‐standardized mortality from hepatobiliary cancers in non‐Hispanic White, aggregated Asian American, and disaggregated Asian American Groups, 2005–2020 National Vital Statistics System.

## Data Availability

Restricted‐use mortality data were obtained from the United States National Center for Health Statistics (NHCS) under a data use agreement. The raw data can be accessed by submitting an application to the NCHS. The population data derived from the American Community Survey were obtained through the Integrated Public Use Microdata Series (IPUMS) through the University of Minnesota and are freely available to the public.
