# Supplementary material for: Disaggregation of Hepatobiliary Cancer Mortality Among Asian Americans: Analysis of NVSS Mortality Data
Source: Cancer Med. 2025 Sep 29;14(19):e71259. doi: 10.1002/cam4.71259 (PMC12477800; doi:10.1002/cam4.71259)
Supplement: Supplementary file 1 — Figure S1: Age‐standardized proportional mortality ratio from hepatobiliary cancers for NHWs, AAs in aggregate, and each AA subgroup. [file CAM4-14-e71259-s005.pdf]

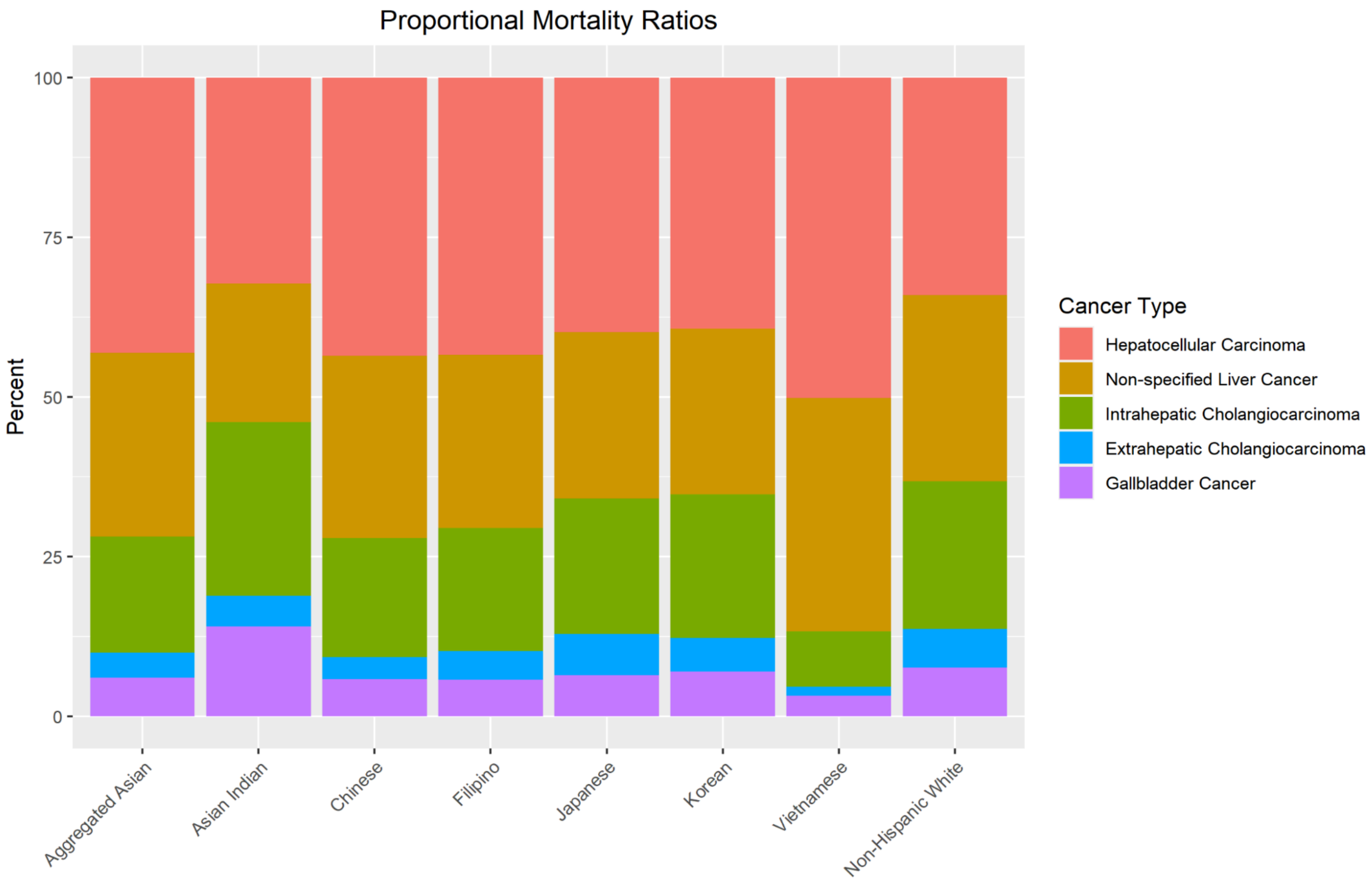

Supplementary Figure 1. Age-standardized proportional mortality ratio from hepatobiliary cancers for NHWs, AAs in aggregate, and each AA subgroup.
