# Supplementary material for: Disaggregation of Hepatobiliary Cancer Mortality Among Asian Americans: Analysis of NVSS Mortality Data
Source: Cancer Med. 2025 Sep 29;14(19):e71259. doi: 10.1002/cam4.71259 (PMC12477800; doi:10.1002/cam4.71259)
Supplement: Supplementary file 3 — Figure S3: Annual age‐adjusted mortality rates (per 100,000) for females from all hepatobiliary cancers, hepatocellular carcinoma, non‐specified liver cancer, intrahepatic cholangiocarcinoma, extrahepatic cholangiocarcinoma, and gallbladder cancers. The data is presented for non‐Hispanic Whites (NHWs) and disaggregated Asian American subgroups for years 2005 to 2020. [file CAM4-14-e71259-s001.pdf]

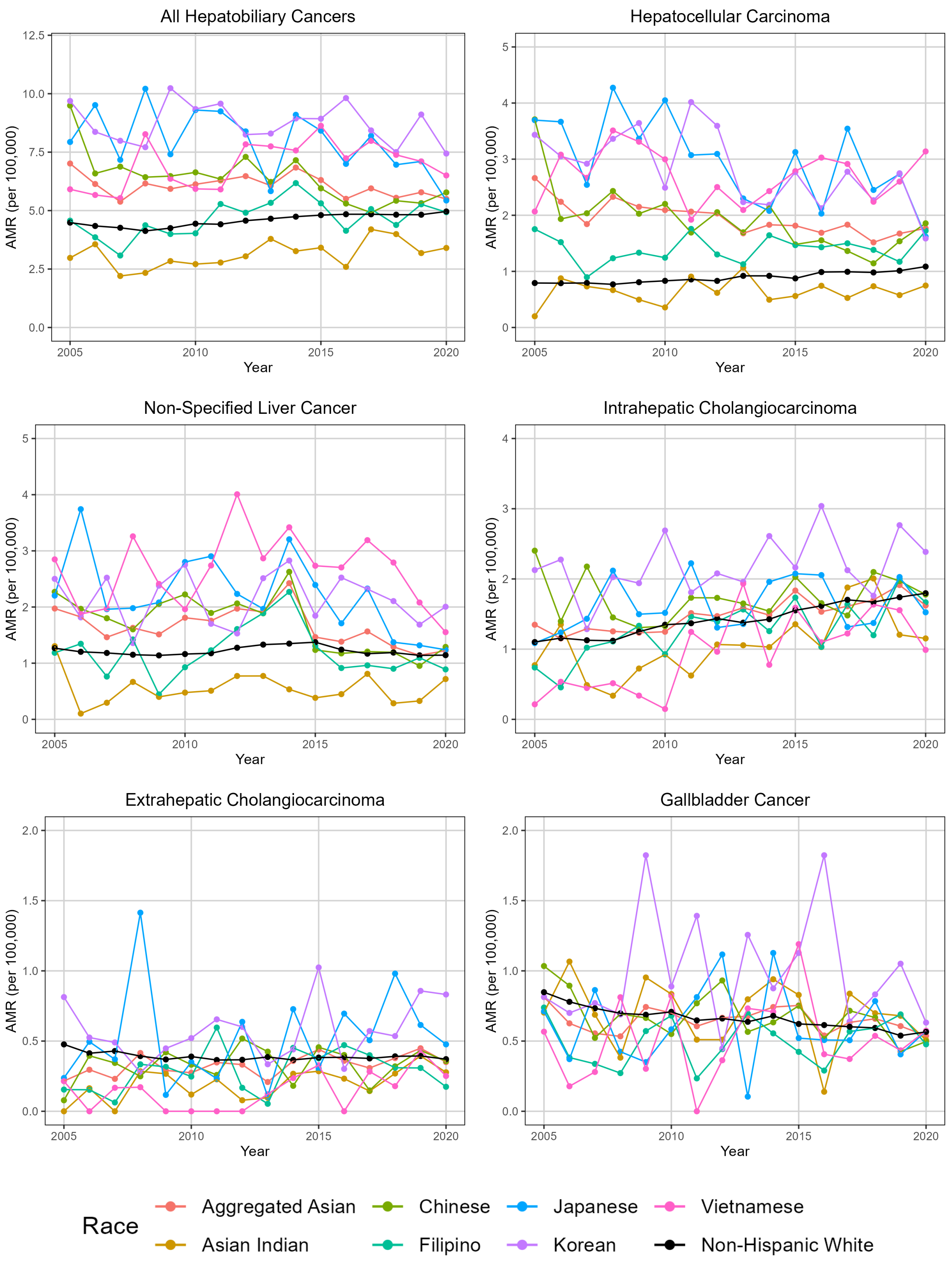
All Hepatobiliary Cancers
Hepatocellular Carcinoma
Non-Specified Liver Cancer
Intrahepatic Cholangiocarcinoma
Extrahepatic Cholangiocarcinoma
Gallbladder Cancer
AMR (per 100,000)
Year
Race
Aggregated Asian
Asian Indian
Chinese
Filipino
Japanese
Korean
Vietnamese
Non-Hispanic White

Supplementary Figure 3. Annual age-adjusted mortality rates (per 100,000) for females from all hepatobiliary cancers, hepatocellular carcinoma, non-specified liver cancer, intrahepatic cholangiocarcinoma, extrahepatic cholangiocarcinoma, and gallbladder cancers. The data is presented for non-Hispanic Whites (NHWs) and disaggregated Asian American subgroups for years 2005 to 2020.
