# Supplementary material for: Disaggregation of Hepatobiliary Cancer Mortality Among Asian Americans: Analysis of NVSS Mortality Data
Source: Cancer Med. 2025 Sep 29;14(19):e71259. doi: 10.1002/cam4.71259 (PMC12477800; doi:10.1002/cam4.71259)
Supplement: Supplementary file 4 — Table S1: Age‐standardized mortality rate (per 100,000 people) and standardized mortality ratios (SMR) from hepatobiliary cancers by race: 2005–2020 National Vital Statistics System. [file CAM4-14-e71259-s003.docx]

**Supplementary Table 1.** Age-standardized mortality rate (per 100,000 people) and standardized mortality ratios (SMR) from hepatobiliary cancers by race: 2005-2020 National Vital Statistics System

| **Panel A: All Sexes** |  |  | **Aggregated Asian** | **Asian Indian** | **Chinese** | **Filipino** | **Japanese** | **Korean** | **Vietnamese** | **Non-Hispanic White** |
| --- | --- | --- | --- | --- | --- | --- | --- | --- | --- | --- |
| **All Hepatobiliary Cancers** |  | **Rate (CI)** | 9.62 (9.44-9.81)* | 3.91 (3.73-4.10)* | 10.86 (10.49-11.26)* | 7.66 (7.36-7.99)* | 8.89 (8.40-9.45)* | 13.79 (12.97-14.74)* | 15.24 (14.44-16.15)* | 6.85 (6.82-6.87) |
|  |  | **SMR (CI)** | 1.40 (1.39-1.42)* | 0.57 (0.55-0.60)* | 1.59 (1.55-1.63)* | 1.12 (1.08-1.16)* | 1.30 (1.22-1.38)* | 2.01 (1.94-2.09)* | 2.23 (2.16-2.30)* | 1 |
| **Hepatocellular Carcinoma** |  | **Rate (CI)** | 4.14 (4.06-4.22)* | 1.26 (1.20-1.32)* | 4.73 (4.56-4.90)* | 3.32 (3.19-3.47)* | 3.54 (3.35-3.76)* | 5.42 (5.10-5.79)* | 7.65 (7.25-8.10)* | 2.33 (2.32-2.34) |
|  |  | **SMR (CI)** | 1.78 (1.74-1.82)* | 0.54 (0.50-0.58)* | 2.03 (1.95-2.11)* | 1.43 (1.35-1.50)* | 1.52 (1.38-1.67)* | 2.33 (2.20-2.46)* | 3.29 (3.14-3.43)* | 1 |
| **Non-specified Liver Cancer** |  | **Rate (CI)** | 2.77 (2.71-2.82)* | 0.85 (0.81-0.89)* | 3.10 (2.99-3.22)* | 2.08 (1.99-2.17) | 2.31 (2.19-2.46)* | 3.58 (3.36-3.83)* | 5.57 (5.27-5.91)* | 2.00 (1.99-2.01) |
|  |  | **SMR (CI)** | 1.38 (1.35-1.42)* | 0.42 (0.38-0.46)* | 1.55 (1.48-1.62)* | 1.04 (0.97-1.11) | 1.16 (1.02-1.30)* | 1.79 (1.67-1.91)* | 2.79 (2.65-2.93)* | 1 |
| **Intrahepatic Cholangiocarcinoma** |  | **Rate (CI)** | 1.75 (1.72-1.78)* | 1.06 (1.02-1.11)* | 2.02 (1.95-2.09)* | 1.47 (1.42-1.53) | 1.89 (1.79-2.01)* | 3.10 (2.92-3.31)* | 1.31 (1.25-1.39)* | 1.58 (1.58-1.59) |
|  |  | **SMR (CI)** | 1.11 (1.07-1.14)* | 0.67 (0.62-0.73)* | 1.28 (1.21-1.35)* | 0.93 (0.86-1.01) | 1.20 (1.05-1.35)* | 1.96 (1.82-2.11)* | 0.83 (0.75-0.92)* | 1 |
| **Extrahepatic Cholangiocarcinoma** |  | **Rate (CI)** | 0.38 (0.37-0.38)* | 0.19 (0.18-0.20)* | 0.38 (0.37-0.40) | 0.35 (0.33-0.36)* | 0.57 (0.54-0.61)* | 0.72 (0.68-0.77)* | 0.22 (0.21-0.23)* | 0.42 (0.42-0.42) |
|  |  | **SMR (CI)** | 0.90 (0.84-0.96)* | 0.45 (0.36-0.54)* | 0.91 (0.80-1.04) | 0.83 (0.70-0.98)* | 1.38 (1.07-1.72)* | 1.73 (1.47-2.00)* | 0.52 (0.39-0.66)* | 1 |
| **Gallbladder Cancer** |  | **Rate (CI)** | 0.59 (0.58-0.60)* | 0.55 (0.52-0.58) | 0.63 (0.61-0.66)* | 0.44 (0.42-0.46)* | 0.57 (0.54-0.61) | 0.97 (0.91-1.04)* | 0.49 (0.47-0.52) | 0.52 (0.52-0.52) |
|  |  | **SMR (CI)** | 1.13 (1.07-1.19)* | 1.05 (0.93-1.18) | 1.21 (1.09-1.34)* | 0.84 (0.73-0.97)* | 1.10 (0.86-1.38) | 1.86 (1.62-2.11)* | 0.94 (0.79-1.11) | 1 |
| **Panel B: Males** |  |  | **Aggregated Asian** | **Asian Indian** | **Chinese** | **Filipino** | **Japanese** | **Korean** | **Vietnamese** | **Non-Hispanic White** |
| **All Hepatobiliary Cancers** |  | **Rate (CI)** | 13.17 (12.92-13.44)* | 4.67 (4.47-4.89)* | 15.33 (14.79-15.91)* | 10.65 (10.20-11.15)* | 9.83 (9.25-10.50) | 18.86 (17.67-20.23)* | 23.52 (22.28-24.90)* | 9.11 (9.08-9.14) |
|  |  | **SMR (CI)** | 1.45 (1.42-1.47)* | 0.51 (0.48-0.54)* | 1.68 (1.63-1.74)* | 1.17 (1.12-1.22)* | 1.08 (0.99-1.18) | 2.07 (1.98-2.16)* | 2.58 (2.49-2.67)* | 1 |
| **Hepatocellular Carcinoma** |  | **Rate (CI)** | 6.33 (6.21-6.46)* | 1.87 (1.80-1.96)* | 7.52 (7.26-7.81)* | 5.24 (5.02-5.48)* | 4.11 (3.86-4.39) | 8.02 (7.52-8.59)* | 12.59 (11.94-13.33)* | 3.77 (3.75-3.78) |
|  |  | **SMR (CI)** | 1.68 (1.64-1.72)* | 0.50 (0.46-0.54)* | 2.00 (1.91-2.09)* | 1.39 (1.30-1.48)* | 1.09 (0.95-1.24) | 2.13 (1.99-2.28)* | 3.34 (3.18-3.51)* | 1 |
| **Non-specified Liver Cancer** |  | **Rate (CI)** | 3.89 (3.81-3.97)* | 1.14 (1.09-1.20)* | 4.49 (4.33-4.66)* | 2.96 (2.83-3.10) | 2.41 (2.27-2.57) | 5.01 (4.69-5.37)* | 8.49 (8.04-9.00)* | 2.78 (2.77-2.79) |
|  |  | **SMR (CI)** | 1.40 (1.35-1.44)* | 0.41 (0.37-0.46)* | 1.61 (1.52-1.70)* | 1.06 (0.98-1.16) | 0.87 (0.72-1.03) | 1.80 (1.65-1.96)* | 3.05 (2.87-3.24)* | 1 |
| **Intrahepatic Cholangiocarcinoma** |  | **Rate (CI)** | 2.01 (1.97-2.05)* | 1.06 (1.02-1.11)* | 2.31 (2.23-2.39)* | 1.66 (1.60-1.74) | 2.15 (2.02-2.29)* | 4.02 (3.76-4.31)* | 1.68 (1.59-1.77) | 1.74 (1.73-1.74) |
|  |  | **SMR (CI)** | 1.16 (1.11-1.21)* | 0.61 (0.54-0.68)* | 1.33 (1.22-1.43)* | 0.96 (0.85-1.07) | 1.24 (1.02-1.48)* | 2.31 (2.09-2.54)* | 0.97 (0.84-1.10) | 1 |
| **Extrahepatic Cholangiocarcinoma** |  | **Rate (CI)** | 0.42 (0.41-0.43) | 0.18 (0.17-0.19)* | 0.43 (0.42-0.45) | 0.41 (0.39-0.43) | 0.63 (0.59-0.67) | 0.87 (0.81-0.93)* | 0.28 (0.27-0.30) | 0.44 (0.44-0.44) |
|  |  | **SMR (CI)** | 0.95 (0.87-1.05) | 0.41 (0.30-0.53)* | 0.97 (0.80-1.16) | 0.92 (0.73-1.15) | 1.42 (0.98-1.96) | 1.95 (1.56-2.38)* | 0.64 (0.45-0.87) | 1 |
| **Gallbladder Cancer** |  | **Rate (CI)** | 0.52 (0.51-0.54)* | 0.41 (0.39-0.43) | 0.59 (0.56-0.61)* | 0.38 (0.37-0.40) | 0.54 (0.51-0.58) | 0.95 (0.89-1.03)* | 0.47 (0.44-0.49) | 0.38 (0.38-0.38) |
|  |  | **SMR (CI)** | 1.39 (1.27-1.51)* | 1.07 (0.88-1.28) | 1.55 (1.32-1.80)* | 1.01 (0.79-1.26) | 1.42 (0.94-2.00) | 2.52 (2.04-3.05)* | 1.23 (0.94-1.56) | 1 |
| **Panel C: Females** |  |  | **Aggregated Asian** | **Asian Indian** | **Chinese** | **Filipino** | **Japanese** | **Korean** | **Vietnamese** | **Non-Hispanic White** |
| **All Hepatobiliary Cancers** |  | **Rate (CI)** | 6.06 (5.95-6.18)* | 3.14 (3.00-3.31)* | 6.39 (6.18-6.61)* | 4.67 (4.51-4.84) | 7.95 (7.56-8.39)* | 8.73 (8.26-9.25)* | 6.97 (6.60-7.39)* | 4.59 (4.57-4.60) |
|  |  | **SMR (CI)** | 1.32 (1.29-1.35)* | 0.69 (0.64-0.73)* | 1.39 (1.33-1.46)* | 1.02 (0.96-1.08) | 1.73 (1.59-1.88)* | 1.90 (1.79-2.02)* | 1.52 (1.43-1.62)* | 1 |
| **Hepatocellular Carcinoma** |  | **Rate (CI)** | 1.95 (1.92-1.99)* | 0.64 (0.61-0.68)* | 1.93 (1.87-2.00)* | 1.41 (1.36-1.46)* | 2.98 (2.83-3.14)* | 2.83 (2.67-3.00)* | 2.71 (2.56-2.87)* | 0.89 (0.89-0.89) |
|  |  | **SMR (CI)** | 2.19 (2.10-2.28)* | 0.72 (0.62-0.84)* | 2.17 (2.00-2.35)* | 1.58 (1.41-1.75)* | 3.34 (2.91-3.81)* | 3.17 (2.85-3.51)* | 3.04 (2.74-3.36)* | 1 |
| **Non-specified Liver Cancer** |  | **Rate (CI)** | 1.65 (1.62-1.68)* | 0.55 (0.52-0.58)* | 1.72 (1.66-1.78)* | 1.20 (1.15-1.24) | 2.21 (2.11-2.34)* | 2.15 (2.03-2.28)* | 2.65 (2.51-2.81)* | 1.22 (1.21-1.22) |
|  |  | **SMR (CI)** | 1.35 (1.29-1.41)* | 0.45 (0.38-0.53)* | 1.41 (1.29-1.53)* | 0.98 (0.87-1.10) | 1.82 (1.54-2.12)* | 1.76 (1.56-1.98)* | 2.18 (1.96-2.41)* | 1 |
| **Intrahepatic Cholangiocarcinoma** |  | **Rate (CI)** | 1.49 (1.46-1.51) | 1.06 (1.01-1.12)* | 1.73 (1.68-1.79)* | 1.28 (1.24-1.33) | 1.63 (1.55-1.72) | 2.19 (2.07-2.32)* | 0.95 (0.90-1.00)* | 1.42 (1.42-1.43) |
|  |  | **SMR (CI)** | 1.04 (0.99-1.10) | 0.75 (0.66-0.84)* | 1.22 (1.12-1.32)* | 0.90 (0.80-1.01) | 1.15 (0.95-1.37) | 1.54 (1.36-1.73)* | 0.67 (0.56-0.79)* | 1 |
| **Extrahepatic Cholangiocarcinoma** |  | **Rate (CI)** | 0.33 (0.32-0.33)* | 0.19 (0.19-0.20)* | 0.33 (0.32-0.34) | 0.29 (0.28-0.30)* | 0.52 (0.49-0.54) | 0.58 (0.55-0.61)* | 0.15 (0.14-0.16)* | 0.39 (0.39-0.39) |
|  |  | **SMR (CI)** | 0.84 (0.76-0.93)* | 0.50 (0.37-0.65)* | 0.85 (0.69-1.02) | 0.73 (0.57-0.92)* | 1.32 (0.93-1.79) | 1.48 (1.15-1.84)* | 0.38 (0.23-0.57)* | 1 |
| **Gallbladder Cancer** |  | **Rate (CI)** | 0.65 (0.64-0.66) | 0.69 (0.66-0.73) | 0.68 (0.65-0.70) | 0.50 (0.48-0.52)* | 0.61 (0.58-0.64) | 0.99 (0.93-1.05)* | 0.52 (0.49-0.55)* | 0.66 (0.66-0.67) |
|  |  | **SMR (CI)** | 0.98 (0.91-1.05) | 1.04 (0.89-1.21) | 1.02 (0.89-1.16) | 0.75 (0.62-0.89)* | 0.92 (0.66-1.22) | 1.48 (1.23-1.76)* | 0.78 (0.61-0.97)* | 1 |

Note. * Indicates that given race group is statistically significant from Non-Hispanic White (p < .05).
