# Supplementary material for: Disaggregation of Hepatobiliary Cancer Mortality Among Asian Americans: Analysis of NVSS Mortality Data
Source: Cancer Med. 2025 Sep 29;14(19):e71259. doi: 10.1002/cam4.71259 (PMC12477800; doi:10.1002/cam4.71259)
Supplement: Supplementary file 5 — Table S2: Average annual percent change (AAPC) in age‐standardized mortality from hepatobiliary cancers for non‐Hispanic White, aggregated Asian American, and disaggregated Asian American groups, 2005–2020 National Vital Statistics System. [file CAM4-14-e71259-s006.docx]

**Supplemental Table 2. Average Annual Percent Change (AAPC) in Age-Standardized Mortality from Hepatobiliary Cancers for Non-Hispanic White, Aggregated Asian American, and Disaggregated Asian American Groups, 2005-2020 National Vital Statistics System**

| **Panel A: All** |  | **Aggregated Asian** | **Asian Indian** | **Chinese** | **Filipino** | **Japanese** | **Korean** | **Vietnamese** | **Non-Hispanic White** |
| --- | --- | --- | --- | --- | --- | --- | --- | --- | --- |
| **All Hepatobiliary Cancers** |  | -0.9 (-1.2, -0.2)* | 2.3 (1.1, 4.4)* | -1.5 (-1.9, -0.6)* | 0.2 (-0.3, 1.5) | -0.9 (-2.1, 0.7) | -1.3 (-2.2, 0.0) | 1.1 (0.8, 1.6)* | 1.2 (1.1, 1.4)* |
| **Hepatocellular Carcinoma** |  | -1.0 (-1.3, -0.5)* | 3.4 (2.0, 5.5)* | -2.5 (-3.5, -1.4)* | -0.9 (-1.6, 0.3) | -2.8 (-3.8, -1.7)* | -2.3 (-3.3, -0.6)* | 1.3 (0.7, 2.5)* | 2.4 (2.0, 2.9)* |
| **Non-specified Liver Cancer** |  | -1.8 (-2.3, -0.5)* | -3.3 (-5.6, 1.9)* | -4.2 (-5.1, -2.5)* | -0.7 (-1.5, 1.0) | -1.1 (-2.8, 3.8) | -1.9 (-2.6, -0.5)* | -0.2 (-0.9, 1.9) | 0.0 (-0.4, 0.3) |
| **Intrahepatic Cholangiocarcinoma** |  | 3.0 (2.1, 4.5)* | 6.9 (4.3, 10.9)* | 2.6 (1.5, 4.8)* | 3.5 (2.1, 5.3)* | 2.9 (1.0, 5.6)* | 1.7 (-0.2, 4.5) | 6.2 (4.0, 9.3)* | 3.5 (3.3, 3.7)* |
| **Extrahepatic Cholangiocarcinoma** |  | 1.2 (-0.3, 3.8) | 2.9 (-0.7, 9.6) | 1.4 (-0.3, 4.9) | -0.8 (-4.0, 3.3) | 0.8 (-3.5, 6.6) | 2.1 (-0.2, 5.4) | 2.5 (-5.0, 10.2) | -0.7 (-1.1, 0.0)* |
| **Gallbladder Cancer** |  | -1.9 (-2.9, 1.7) | -3.1 (-6.4, 3.2) | -4.5 (-5.7, -2.8)* | -0.3 (-2.2, 5.0) | 0.8 (-3.1, 6.0) | -1.9 (-4.8, 4.6) | 2.8 (-0.3, 7.7) | -2.0 (-2.5, -1.5)* |
| **Panel B: Males** |  | **Aggregated Asian** | **Asian Indian** | **Chinese** | **Filipino** | **Japanese** | **Korean** | **Vietnamese** | **Non-Hispanic White** |
| **All Hepatobiliary Cancers** |  | -0.3 (-0.5, 0.3) | 4.7 (3.2, 8.2)* | -1.4 (-1.8, 0.1) | -0.7 (-1.2, 0.4) | 1.0 (0.0, 2.3) | -1.2 (-2.3, 0.5) | 1.3 (0.9, 2.2)* | 1.7 (1.6, 1.9)* |
| **Hepatocellular Carcinoma** |  | -0.8 (-1.1, -0.1)* | 4.2 (2.4, 6.9)* | -2.3 (-3.0, -0.8)* | -1.7 (-2.8, -0.3)* | -0.3 (-2.5, 2.5) | -1.3 (-2.8, 1.6) | 1.5 (0.6, 2.8)* | 2.3 (2.0, 2.8)* |
| **Non-specified Liver Cancer** |  | -1.5 (-1.9, -0.8)* | -0.8 (-3.5, 6.6) | -4.2 (-5.5, -1.7)* | -1.1 (-1.9, 2.6) | 1.6 (-1.1, 5.3) | -2.4 (-3.5, -0.5)* | 0.2 (-0.4, 1.3) | 0.3 (-0.1, 0.7) |
| **Intrahepatic Cholangiocarcinoma** |  | 3.8 (2.9, 5.5)* | 9.1 (6.4, 13.5)* | 4.1 (1.9, 9.0)* | 3.0 (0.9, 5.9)* | 4.9 (1.4, 10.4)* | 1.8 (-0.7, 5.5) | 6.7 (4.2, 11.1)* | 3.5 (3.3, 3.8)* |
| **Extrahepatic Cholangiocarcinoma** |  | 0.8 (-1.2, 4.2) | -3.5 (-8.1, 3.8) | 2.6 (0.5, 10.9)* | -0.3 (-3.4, 5.2) | -2.7 (-6.5, 2.8) | 0.5 (-3.9, 8.6) | 8.4 (4.5, 16.4)* | 0.2 (-0.4, 0.8) |
| **Gallbladder Cancer** |  | -2.3 (-4.3, 5.0) | 4.8 (0.5, 16.5)* | -5.0 (-8.3, 0.2) | -6.7 (-4.1, 24.3) | -0.8 (-6.2, 7.8) | -5.8 (-10.3, 19.3) | 0.6 (-2.3, 5.8) | -1.6 (-2.2, -0.9)* |
| **Panel C: Females** |  | **Aggregated Asian** | **Asian Indian** | **Chinese** | **Filipino** | **Japanese** | **Korean** | **Vietnamese** | **Non-Hispanic White** |
| **All Hepatobiliary Cancers** |  | -1.0 (-1.6, -0.2)* | 1.7 (0.5, 3.5)* | -1.7 (-2.3, -0.5)* | 1.3 (0.1, 3.3)* | -3.1 (-4.0, -0.7)* | -1.0 (-1.9, 2.4) | 1.1 (0.2, 2.8)* | 0.6 (0.5, 0.8)* |
| **Hepatocellular Carcinoma** |  | -2.0 (-2.7, -1.2)* | 2.1 (-0.3, 7.7) | -2.6 (-4.0, -0.9)* | 2.1 (0.6, 5.1)* | -5.4 (-7.8, -2.6)* | -4.9 (-6.7, 2.7) | 1.5 (0.4, 4.8)* | 1.9 (1.6, 2.5)* |
| **Non-specified Liver Cancer** |  | -2.1 (-3.1, 1.5) | 5.4 (1.4, 13.1)* | -4.0 (-5.5, -0.5)* | -3.2 (-7.2, 3.5) | -4.4 (-6.3, 0.8) | -0.4 (-2.1, 2.7) | -4.6 (-7.4, -1.7)* | -0.7 (-1.2, -0.3)* |
| **Intrahepatic Cholangiocarcinoma** |  | 1.9 (1.1, 3.1)* | 5.7 (3.2, 14.5)* | 0.7 (-0.8, 3.2) | 4.6 (2.7, 7.7)* | 3.3 (-1.9, 10.5) | 1.5 (0.1, 4.0)* | 8.7 (4.9, 25.2)* | 3.5 (3.0, 3.9)* |
| **Extrahepatic Cholangiocarcinoma** |  | 1.9 (0.1, 4.8)* | 6.0 (1.7, 14.2)* | 2.1 (-0.7, 7.2) | 0.2 (-5.2, 7.5) | 3.3 (-2.0, 10.5) | 4.6 (2.2, 9.7)* | 2.9 (0.9, 8.9)* | -1.3 (-1.8, -0.4)* |
| **Gallbladder Cancer** |  | -1.7 (-2.9, 0.1) | -2.5 (-6.6, 4.9) | -3.3 (-5.1, 0.1) | 1.1 (-2.1, 6.2)* | 0.3 (-4.5, 7.2) | -2.7 (-6.8, 3.5) | 1.7 (-1.4, 9.1) | -2.7 (-3.1, -2.0)* |

Note. * p < .05
