# Supplementary material for: Disaggregation of Hepatobiliary Cancer Mortality Among Asian Americans: Analysis of NVSS Mortality Data
Source: Cancer Med. 2025 Sep 29;14(19):e71259. doi: 10.1002/cam4.71259 (PMC12477800; doi:10.1002/cam4.71259)
Supplement: Supplementary file 6 — Table S3: Annual percent change (APC) joinpoint regression results of age‐standardized mortality from hepatobiliary cancers in non‐Hispanic White, aggregated Asian American, and disaggregated Asian American Groups, 2005–2020 National Vital Statistics System. [file CAM4-14-e71259-s002.docx]

Supplemental Table 3. Annual Percent Change (APC) Joinpoint Regression Results of Age-Standardized Mortality from Hepatobiliary Cancers in Non-Hispanic White, Aggregated Asian American, and Disaggregated Asian American Groups, 2005-2020 National Vital Statistics System

|  | All Hepatobiliary Cancers | Hepatocellular Carcinoma | Non-specified Liver Cancer | Intrahepatic Cholangiocarcinoma | Extrahepatic Cholangiocarcinoma | Gallbladder Cancer |
| --- | --- | --- | --- | --- | --- | --- |
|  | Year Interval: APC (95% CI) | Year Interval: APC (95% CI) | Year Interval: APC (95% CI) | Year Interval: APC (95% CI) | Year Interval: APC (95% CI) | Year Interval: APC (95% CI) |
| Aggregated Asian Americans |  |  |  |  |  |  |
| All | 2005-2007: -5.6 (-9.0, 0.7)  2007-2013: 1.8 (02.2, 5.5)  2013-2020: -1.9 (-2.4,-1.0)* | 2005-2020: -1.0 (-1.3, -0.5)* | 2005-2014: 1.5 (-0.2, 5.4)  2014-2020: -6.5 (-8.6, -4.1)* | 2005-2020: 3.0 (2.1, 4.5)* | 2005-2020: 1.2 (-0.3, 3.8) | 2005-2018: 0.5 (-1.6, 25.1)  2018-2020: -16.0 (-22.2, -2.3)* |
| Male | 2005-2013: 0.9 (0.1, 4.6)*  2013-2020: -1.6 (-3.1, -0.8)* | 2005-2020: -0.8 (-1.1, -0.1)* | 2005-2015: 1.6 (0.3, 3.1)*  2015-2018: -12.2 (-15.3, 1.0)  2018-2020: -0.2 (-5.4, 5.1) | 2005-2020: 3.8 (2.9, 5.5)* | 2005-2020: 0.8 (-1.2, 4.2) | 2005-2018: 1.8 (-5.7, 82.1)  2018-2020: -25.1 (-35.9, -1.2) |
| Female | 2005-2020: -1.0 (-1.6, -0.2)* | 2005-2018: -2.9 (-8.4, -1.8)*  2018-2020 (4.5 (-1.3, 9.8) | 2005-2014: 1.3 (-1.4, 27.9)  2014-2020: -7.0 (-12.8, -3.2)* | 2005-2020: 1.9 (1.1, 3.1)* | 2005-2020: 1.9 (0.1, 4.8)* | 2005-2020: -1.7 (-2.9, 0.1) |
| Asian Indian |  |  |  |  |  |  |
| All | 2005-2020: 2.3 (1.1, 4.4)* | 2005-2020: 3.4 (2.0, 5.5)* | 2005-2007: -34.1 (-52.2, 8.2)  2007-2013: 15.6 (-14.7, 46.5)  2013-2020: -7.4 (-11.7, 2.5) | 2005-2020: 6.9 (4.3, 10.9)* | 2005-2020: 2.9 (-0.7, 9.6) | 2005-2020: -3.1 (-6.4, 3.2) |
| Male | 2005-2011: 11.1 (5.1, 43.0)*  2011-2020: 0.6 (-4.2, 3.0) | 2005-2020: 4.2 (2.4, 6.9)* | 2005-2015: 7.8 (1.2, 60.3)*  2015-2020: -16.1 (-30.7, -5.4)* | 2005-2020: 9.1 (6.4, 13.5)* | 2005-2020: -3.5 (-8.1, 3.8) | 2005-2010 40.3 (10.8, 208.3)*  2010-2020: -9.4 (-15.4, -3.4)* |
| Female | 2005-2020: 1.7 (0.5, 3.5)* | 2005-2020: 2.1 (-0.3, 7.7) | 2005-2020: 5.4 (1.4, 13.1)* | 2005-2018: 9.6 (-2.8, 99.1)  2018-2020: -16.7 (-27.6, 8.2) | 2005-2020: 6.0 (1.7, 14.2)* | 2005-2020: -2.5 (-6.6, 4.9) |
| Chinese |  |  |  |  |  |  |
| All | 2005-2014: -1.1 (-2.2, 4.6)  2014-2018: -6.9 (-11.5, -3.7)*  2018-2020: 8.0 (2.5, 13.8)* | 2005-2018: -3.8 (-9.9, -2.4)*  2018-2020: 6.4 (-1.4, 13.4) | 2005-2020: -4.2 (-5.1, -2.5)* | 2005-2020: 2.6 (1.5, 4.8)* | 2005-2020: 1.4 (-0.3, 4.9) | 2005-2020: -4.5 (-5.7, -2.8) |
| Male | 2005-2013: -0.3 (-1.8, 7.5)  2013-2018: -6.4 (-13.4, -3.6)*  2018-2020: 7.6 (2.1, 15.7)* | 2005-2020: -2.3 (-3.0, -0.8) | 2005-2020: -4.2 (-5.5, -1.7) | 2005-2020: 4.1 (1.9, 9.0)* | 2005-2010: 9.0 (-1.8, 116.4)  2010-2016: -10.3 (-32.5, -3.2)*  2016-2020: 16.5 (6.7, 59.2)* | 2005-2020: -5.0 (-8.3, 0.2) |
| Female | 2005-2020: -1.7 (-2.3, -0.5)* | 2005-2018: -5.6 (-10.7, -3.3)  2018-2020: 19.2 (3.2, 32.5) | 2005-2020: -3.9 (-5.5, -0.5)* | 2005-2020: 0.7 (-0.7, 3.3) | 2005-2020: 2.1 (-0.7, 7.2) | 2005-2020: -3.3 (-5.1, 0.1) |
| Filipino |  |  |  |  |  |  |
| All | 2005-2015: 2.1 (0.8, 7.0)*  2015-2020: -3.4 (-7.0, -1.0)* | 2005-2020: -0.9 (-1.6, 0.3) | 2005-2014: 3.5 (1.1, 9.4)*  2014-2020: -6.9 (-10.0, -3.7)* | 2005-2020: 3.5 (2.1, 5.3)* | 2005-2020: -0.8 (-4.0, 3.3) | 2005-2018: 2.5 (-7.9, 53.7)  2018-2020: -17.0 (-26.0, 1.5) |
| Male | 2005-2018: 0.8 (-0.1, 3.4)  2018-2020: -10.0 (-13.2, -2.3)* | 2005-2020: -1.7 (2.8, -0.2)* | 2005-2015: 1.8 (-0.4, 29.6)  2015-2020: -6.6 (-11.8, -2.1)* | 2005-2020: 3.0 (0.9, 5.9)* | 2005-2020: -0.3 (-3.4, 5.2) | 2005-2007: 131.1 (-10.2, 598.8)  2007-2018: -0.1 (-19.7, 33.8)  2018-2020: -29.1 (-39.2, -1.2)* |
| Female | 2005-2020: 1.3 (0.1, 3.3)* | 2005-2020: 2.1 (0.6, 5.1)* | 2005-2009: -17.8 (-51.5, 15.9)  2009-2012:40.4 (-27.1, 72.5)  2012-2020: -8.6 (-16.5, 18.1) | 2005-2020: 4.6 (2.7, 7.7)* | 2005-2020: 0.2 (-5.2, 7.5) | 2005-2020: 1.1 (-2.1, 6.2) |
| Japanese |  |  |  |  |  |  |
| All | 2005-2020: -0.9 (-2.1, 0.7) | 2005-2014: -3.3 (-12.0, -1.0)*  2014-2017: 11.8 (0.9, 18.7)*  2017-2020: -14.0 (-21.3, -7.4)* | 2005-2014: 2.3 (-5.0, 51.2)  2014-2020: -6.1 (-15.7, 2.6) | 2005-2020: (2.9 (1.0, 5.6)* | 2005-2020: 0.8 (-3.5, 6.6) | 2005-2020: 0.8 (-3.1, 6.0) |
| Male | 2005-2020: 1.0 (-0.2, 2.3) | 2005-2020: -0.3 (-2.5, 2.5) | 2005-2020: 1.6 (-1.1, 5.3) | 2005-2020: 4.9 (1.4, 10.4)* | 2005-2020:-2.7 (-6.5, 2.8) | 2005-2020: -0.8 (-6.2, 7.8) |
| Female | 2005-2018: -1.0 (-3.0, 15.0)  2018-2020: -15.4 (-21.6, -3.1)* | 2005-2020: -5.4 (-7.8, -2.6)* | 2005-2014: 0.7 (-4.2, 48.1)  2014-2020: -11.4 (-24.0, -4.8) | 2005-2020: 0.5 (-1.9, 3.6) | 2005-2020: 3.3 (-1.9, 10.5) | 2005-2020 (0.3, -4.5, 7.2) |
| Korean |  |  |  |  |  |  |
| All | 2005-2020: -1.3 (-2.2, 0.2) | 2005-2020: -2.3 (-3.3, -0.6)* | 2005-2018: -1.9 (-2.6, -0.5)* | 2005-2020: 1.7 (-0.2, 4.5) | 2005-2020: 2.1 (-0.2, 5.4) | 2005-2016: 6.8 (0.3, 56.0)*  2016-2020: -22.2 (-42.1, -7.2)* |
| Male | 2005-2020: -1.2 (-2.3, 0.5) | 2005-2020: -1.3 (-2.8, 1.6) | 2005-2020: -2.4 (-3.5, -0.5)* | 2005-2020: 1.8 (-0.6, 5.5) | 2005-2020: 0.5 (-3.9, 8.6) | 2005-2018: 3.2 (-6.8, 345.4)  2018-2020: -47.9 (-61.2, -5.8) |
| Female | 2005-2016: 0.3 (-3.6, 30.7)  2016-2020: -4.6 (-10.4, 0.9) | 2005-2018: -2.5 (-11.4, 76.8)  2018-2020: -19.1 (-27.9, -1.0)* | 2005-2020: -0.4 (-2.1, 2.7) | 2005-2020: 1.5 (0.1, 4.0)* | 2005-2020: 4.6 (2.2, 9.7)* | 2005-2020: -2.7 (-6.8, 3.5) |
| Vietnamese |  |  |  |  |  |  |
| All | 2005-2018: 2.5 (1.9, 3.6)*  2018-2020: -7.6 (-9.7, -2.6)* | 2005-2020: 1.3 (0.7, 2.5)* | 2005-2014: 5.9 (2.9, 12.3)*  2014-2020: -8.7 (-11.7, -4.9) | 2005-2020: 6.2 (4.0, 9.3)* | 2005-2010: -16.7 (-52.6, 5.7)  2010-2020: 13.7 (9.9, 44.1) | 2005-2020: 2.8 (-0.3, 7.7) |
| Male | 2005-2018: 2.6 (1.7, 4.9)*  2018-2020: -6.8 (-9.6, -0.1)* | 2005-2020: 1.5 (0.6, 2.8)* | 2005-2014: 5.9 (3.8, 8.7)*  2015-2020: -7.6 (-9.5, -5.3)* | 2005-2020: 6.8 (4.2, 11.1)* | 2005-2020: 8.4 (4.5, 16.4)* | 2005-2020: 0.6 (-2.3, 5.8) |
| Female | 2005-2015: 3.9 (1.9, 12.6)*  2015-2020: -4.3 (-10.8, -0.9) | 2005-2020: 1.5 (0.3, 4.8)* | 2005-2017: 3.2 (0.1, 10.7)*  2017-2020: -21.8 (-31.5, -9.0)* | 2005-2018: 15.2 (-0.6, 251.1)  2018-2020: -25.2 (-39.1, 12.7) | 2005-2020: 2.9 (0.8-8.9)* | 2005-2020: 1.7 (-1.4, 9.1) |
| Non-Hispanic White |  |  |  |  |  |  |
| All | 2005-2007: -0.5 (-1.4, 1.1)  2007-2014: 2.8 (2.5, 3.2)*  2014-202: 0.1 (-0.2, 0.4) | 2005-2011: 3.3 (2.5 (7.7)*  2011-2020: (1.8 (0.2, 2.2)* | 2005-2010: 0.8 (-2.3, 2.1)  2010-2013: 7.6 (4.2, 9.3)*  2013-2020: -3.7 (-4.5, -3.0)* | 2005-2020: 3.5 (3.3, 3.7)* | 2005-2009: -4.3 (-10.1, -1.2)*  2009-2020: 0.7 (0.1, 2.1)* | 2005-2020: -2.0 (-2.5, -1.5)* |
| Male | 2005-2014: 3.0 (2.6, 3.5)*  2014-2020: -0.1 (-0.8, 0.5) | 2005-2010: 3.9 (1.5, 7.4)*  2010-2017: 2.1 (0.7, 5.4)*  2017-2020: 0.3 (-2.0, 1.9) | 2005-2010: 2.1 (-2.0, 3.7)  2010-2013: 8.1 (4.2, 9.9)  2013-2020: -4.1 (-4.9, -3.3) | 2005-2020: 3.5 (3.3, 3.8)* | 2005-2013: -1.5 (-5.1, -0.2)*  2013-2020: 2.2 (0.9, 5.9)* | 2005-2020: -1.6 (-2.2, -0.9)* |
| Female | 2005-2008: -2.3 (-4.1, -1.0)*  2008-2014: 2.3 (1.8, 3.1)*  2014-2020: 0.5 (-0.3, 0.9) | 2005-2008: -0.6 (-4.5, 2.4)  2008-2020: 2.6 (1.6, 4.8)* | 2005-2009: -2.7 (-7.8, -0.3)*  2009-2014: 3.9 (2.2, 7.2)*  2014-2020: -3.2 (-4.5, -2.1)* | 2005-2020: 3.5 (3.0, 3.9)* | 2005-2009: -5.1 (-11.4, -1.4)*  2009-2020: 0.2 (-0.5, 2.7) | 2005-2007: -7.2 (-10.5, -2.3)*  2007-2020: -2.0 (-2.4, -0.4)* |

Note. * p < .05
